# A New Gas Analysis Method Based on Single-Beam Excitation Stimulated Raman Scattering in Hollow Core Photonic Crystal Fiber Enhanced Raman Spectroscopy

**DOI:** 10.3390/bioengineering10101161

**Published:** 2023-10-03

**Authors:** Maryam Shirmohammad, Michael A. Short, Haishan Zeng

**Affiliations:** 1 Department of Physics and Astronomy, University of British Columbia, 6224 Agricultural Road, Vancouver, BC V6T 1Z1, Canada; smaryam@umich.edu; 2 Imaging Unit, Integrative Oncology Department, BC Cancer Research Institute, Vancouver, BC V5Z 1L3, Canada; mshort@bccrc.ca; 3 Department of Dermatology and Skin Science, University of British Columbia, Vancouver, BC V5Z 4E8, Canada

**Keywords:** stimulated Raman scattering, gas Raman spectroscopy, hollow core photonic crystal fiber, Raman enhancement technique

## Abstract

We previously developed a hollow-core photonic crystal fiber (HCPCF) based Raman scattering enhancement technique for gas/human breath analysis. It enhances photon–gas molecule interactions significantly but is still based on CW laser excitation spontaneous Raman scattering, which is a low-probability phenomenon. In this work, we explored nanosecond/sub-nanosecond pulsed laser excitation in HCPCF based fiber enhanced Raman spectroscopy (FERS) and successfully induced stimulated Raman scattering (SRS) enhancement. Raman measurements of simple and complex gases were performed using the new system to assess its feasibility for gas analysis. We studied the gas Raman scattering characteristics, the relationship between Raman intensities and pump energies, and the energy threshold for the transition from spontaneous Raman scattering to SRS. H_2_, CO_2_, and propene (C_3_H_6_) were used as test gases. Our results demonstrated that a single-beam pulsed pump combined with FERS provides an effective Raman enhancement technique for gas analysis. Furthermore, an energy threshold for SRS initiation was experimentally observed. The SRS-capable FERS system, utilizing a single-beam pulsed pump, shows great potential for analyzing complex gases such as propene, which is a volatile organic compound (VOC) gas, serving as a biomarker in human breath for lung cancer and other human diseases. This work contributes to the advancement of gas analysis and opens alternative avenues for exploring novel Raman enhancement techniques.

## 1. Introduction

Raman spectroscopy is a versatile analytical technique commonly used for gas analysis. Raman analyzers based on spontaneous Raman scattering have been developed as gas sensors for a variety of applications. Utilization of Raman enhancement techniques is an indispensable part of Raman gas analysis systems as spontaneous Raman scattering is intrinsically an inefficient phenomenon. Increasing the effective pathlength for Raman interaction with multiple laser passing is a common enhancement technique that is achieved by using specialty mirrors or prisms in the gas cell [1,2,3,4]. Raman gas analyzers based on spontaneous Raman scattering have been developed in petrochemical industries [5,6], environmental studies [7,8], and more demanding fields such as analysis of exhaled breath [9,10,11,12].

Fiber enhanced Raman spectroscopy (FERS) [13] is relatively a new enhancement technique for gas analysis that utilizes specialized micro-structured hollow-core optical fibers that are designed to have minimal attenuation losses. These micro-structured optical fibers fall into the category of either band-gap transmission hollow-core fibers [14,15] or anti-resonant hollow-core fibers [16,17]. Light is confined to the core of these fibers and, therefore, the probability of light–matter interaction including Raman interactions in the hollow core increases considering the micron size scale of the core [18]. Light transmission through these fibers is maximum when the excitation pump wavelength falls within the transmission bandwidth of the fiber. Therefore, increasing the physical length of the fiber will result in increasing the light–gas interaction pathlength without significant attenuation of Raman signals. FERS systems incorporating band-gap fibers such as hollow-core photonic crystal fibers (HCPCFs) [10,19,20] or ant-resonant hollow-core fibers [21,22,23,24,25] are reported with an increasing trend in the literature for sensitive gas analysis applications.

Many reports have shown that HCPCFs are highly efficient for an enhancement in spontaneous Raman scattering intensities for analytical applications [10,20,26,27,28]. Continuous wave (CW) excitation sources are generally used with FERS systems for spectroscopy applications. Due to the Raman-enhancing mechanism of HCPCFs, FERS-based gas analyzers have been developed for demanding applications such as the analysis of trace amounts of gas components in petrochemical industries [27,28,29] and breath analysis for disease monitoring and diagnosis [10,19,30,31]. Our group is one of the two pioneers [10,30] who applied FERS for breath analysis using CW laser-excited spontaneous Raman scattering.

Stimulated Raman scattering (SRS) is an efficient coherent Raman enhancement technique capable of providing Raman spectra of different mediums with orders of magnitude improvements to Raman signal intensity over spontaneous Raman scattering. SRS spectroscopy is generally performed with the use of two laser beams as a pump and Stokes probe, where the difference in frequency between the two beams matches the vibrational frequency in the gas sample, and hence, the molecular vibrations are coherently driven [32]. The SRS process can be achieved by using a single-beam pump excitation source as well, with a high-power intensity. Pulsed lasers with relatively high peak power can be used as excitation sources for SRS [33,34].

The advent of HCPCFs has led to the realization of the SRS process with much smaller energy thresholds that are not achievable using CW lasers. For instance, hydrogen-filled HCPCFs have been pumped with single-beam nanosecond excitation-pulsed lasers, and SRS has been generated from pump pulses with peak powers as low as ~100 W [33,34,35].

Although HCPCFs with pulsed pumps have been reported for SRS and other nonlinear optics application [36] and with CW pumps for gas analysis applications (e.g., FERS systems), to the best of our knowledge, the combination of a single-beam high-energy pulsed pump-generated SRS and FERS geared toward gas analysis has not been reported. Single-beam SRS offers great advantages, such as being simple, cost-effective, and fast, and has great potential for improving gas analysis. Therefore, a feasibility study on the application of a single-beam pulsed excitation pump with an FERS system for the analysis of gases was the main motivation for the current study.

In spontaneous Raman scattering, the intensity of the Raman signal has a linear dependence on excitation pump power, as shown in Equation (1)

(1) $$I\left(\omega_{s}\right)=I_{p}Nz\sigma \left(\omega_{s}\right)$$

where $I\left(\omega_{s}\right)$ is the Raman scattering intensity, $I_{p}$ is the pump intensity represented by the power of the excitation pump, *N* is the molecular number density of target gas molecules, z is the light pathlength in the target gas, σ is the Raman scattering cross-section, and $\omega_{s}$ is the oscillation frequency. The literature has theoretically shown that the SRS process initiates when the energy in the excitation pump reaches a certain threshold [37], beyond which the Stokes intensity increases exponentially with pump power, and hence, a drastic Raman scattering intensity enhancement.

Despite numerous published studies on HCPCF gas sensing with spontaneous Raman scattering, the properties of the SRS signal of molecular vibrations and or rotations in HCPCF are not yet fully explored. This study aims at (1) exploring the feasibility of gas analysis based on SRS in an FERS system pumped with a single-beam pulsed laser, (2) studying the dependency of the Raman scattering intensity on the pulsed pump energy and the presence of an SRS energy threshold, and (3) further assessing the potential of the single-beam pulsed laser-excited, SRS-capable FERS system in spectral analysis of complicated gases with industrial and biomedical association.

## 2. Materials and Methods

### 2.1. Overview of the FERS System Using a Single-Beam Pulsed Pump

**Figure 1:**
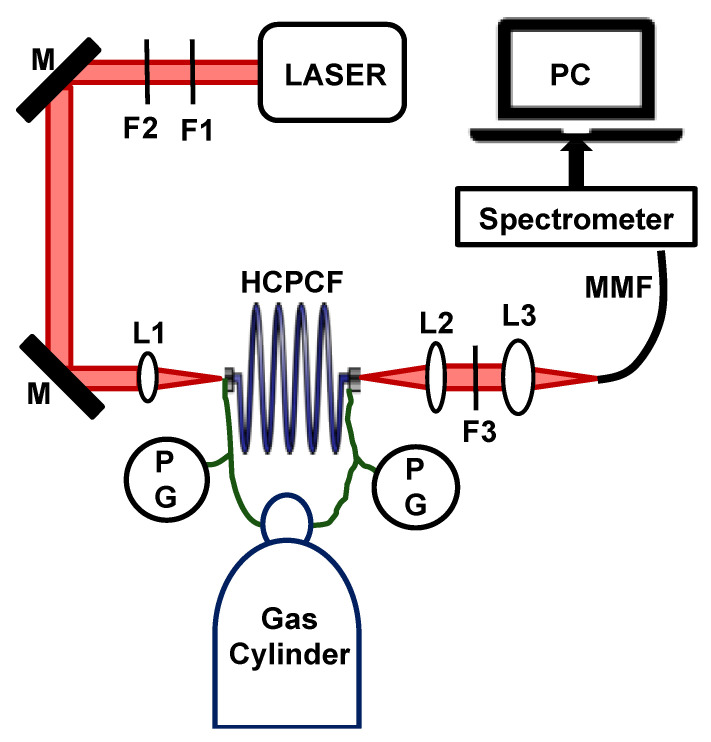
The main components of the FERS gas analysis system include a high-energy pulsed laser, an HCPCF, and a spectrometer. Additional optical components include F1: 785 nm laser line filter, F2: neutral density filter, M: mirror, PG: pressure gauge, L1–L3: lenses, F3: 785 nm long pass filter, MMF: 50 µm multimode fiber.

A schematic diagram of the FERS system for SRS measurement of gases is shown in Figure 1. The system is modified from our previous system described in Chow et al. [10], where the CW excitation laser was replaced with a pulsed-pump laser for the realization of SRS. We tried SRS measurements using two different high-energy pulsed lasers: an optical parametric oscillator (OPO)-based laser (Opolette 355 LD, Opotek, LLC, Carlsbad, CA, USA) and a dye laser pumped with a 337 nm nitrogen laser (GL-302, Horiba Scientific, Irvine, CA, USA). The OPO laser had a pulse duration of 7 ns, a pulse repetition rate of 20 Hz, and a bandwidth of 4–7 cm^−1^, and the dye laser had a pulse duration of 0.8 ns, with a pulse repetition rate of 7 Hz, and a spectral bandwidth of 0.7 cm^−1^. To obtain a 785 nm output from the dye laser, oxazine 750 perchlorate dye (OD 775, Exciton) was chosen as the fluorescent dye. The output of the lasers was tuned to 785 nm for Raman measurements. Of note is that the OPO laser had the advantage of being compact (30.5 cm × 17.8 cm × 12.4 cm size) and easier to work with compared with the dye laser, which had bulky components (system size: 128.5 cm × 51.5 cm × 23.5 cm).

The other main component of the FERS system is an HCPCF (HC-800-B, NKT Photonics) with a length of 2.5 m, core diameter of 7.5 ± 1 µm, and spectral transmission window of 770–870 nm with an attenuation of ≤ 150 dB/km. The Raman photons were collected in forward scattered mode and were transferred to a spectrometer using a 50 µm multimode fiber.

All gas measurements were performed at room temperature. Prior to Raman measurements, the HCPCF was evacuated using a vacuum pump first, and then pressurized with the sample gas. The pressure of the system was monitored at the two ends of the HCPCF using pressure gauges (PGs). The pump pulses passed through a 785 nm laser line filter (F1; LL01-785-12.5, Semrock, Rochester, NY, USA), and then a neutral density filter (F2) for beam energy adjustments. The beam was focused on the HCPCF and collected from the output end using aspheric lenses (L1 and L2, f = 13.86 mm; C560TME-B, Thorlabs, Newton, NJ, USA). The emitted beam passed through a long pass filter (F3; LP02-785RU, Semrock, Rochester, NY, USA) for transmitting Stokes Raman pulses, and finally, was focused into a multimode fiber of the spectrometer using a third aspheric lens (L3, f = 18.4 mm; C280TMD-B, Thorlabs) for spectral analysis. The spectrometer consists of an Imaging Spectrograph (HoloSpec™ f/2.2, Kaiser Optical Systems, Ann Arbor, MI, USA) and a CCD camera (Apogee Camera, Alta series, Oxford Instruments Inc., Concord, MA, USA).

### 2.2. Description of Gas Raman Measurements

In this work, feasibility studies of the FERS system pumped with pulsed lasers were performed with H_2_ and CO_2_ with well-known Raman characteristics [33,34,38,39,40]. Propene and some of its derivatives are frequently found in breath and are biomarkers of lung cancers [41,42]. Therefore, as a more complicated volatile organic compound (VOC) gas, propene was used for studying the performance of the single-beam pulsed-laser pump-excited FERS in the analysis of VOCs for potential cancer detection applications.

*H_2_ Raman measurements:* Raman experiments were performed with H_2_ at a pressure of 110 psi with both the OPO and the dye laser with gradually increasing pulse energies. The pulse energy of the OPO varied from 60 nJ to 5 µJ and that of the dye laser varied from 15 nJ to 1 µJ. Raman intensity-laser pulse energy data were obtained and analyzed.

*CO_2_ Raman measurements:* Raman measurements were performed with CO_2_ at a pressure of 120 psi. The OPO pump pulse energies varied from 0.7 to 7 µJ and that of the dye laser varied from 50 nJ to 3.5 µJ.

*Propene Raman measurements*: Raman measurements were obtained from propene at a pressure of 100 psi with the dye laser pulses as the pump. The measurements were performed with pump energies varying from 1 to 4 µJ.

It should be mentioned that at a given pulse energy level, the dye laser has almost an order of magnitude higher power density compared with the OPO because the pulse widths of the two lasers have an 8.8 times difference (0.8 ns/7 ns = 8.8). We hope that by combining experiments with the two lasers that span a larger light power density range, it will be more likely to observe the transition from spontaneous Raman to SRS as the pump laser pulse energy varies.

### 2.3. Raman Spectral Analysis

All the samples investigated in this study are pure gases from purchased gas tanks, and we studied one sample for each gas type. Each Raman spectrum was the average of five Raman measurements after being normalized to the spectrometer integration time and calibrated to Raman wavenumbers. Integration times varied from 1 s to 300 s depending on the intensity of the Raman signals. For example, the required integration time was in the order of seconds for the spectra with strong SRS peaks. However, for less intense Raman spectra, e.g., under small pulse energy excitation, the integration times were set to multiple hundred seconds to minimize the background noise. Raman intensities were calculated as the area of the Raman signal peak following background subtraction. Error bars were calculated as the standard error of the measured Raman intensities. Raman measurements with different pump pulse energies were obtained to investigate Raman intensity changes with varying pump pulse energies.

## 3. Results

### 3.1. H_2_ Raman Measurements

**Figure 2:**
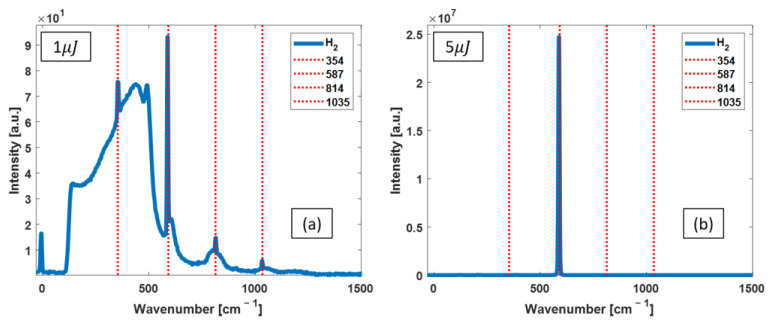
Raman spectra of the H_2_-filled FERS system pumped with OPO pulses with two different energies of 1 µJ (**a**) and 5 µJ (**b**). The red dashed lines identify the known peak positions of rotational Raman lines of H_2_. The spectrum acquired at 1 µJ shows an attenuated pump laser line at 0 cm^−1^, along with a broad emission from about 100 to 500 cm^−1^, and a less intense broad profile with maxima located around 600, 800, and 1050 cm^−1^, which are due to Raman scattering from the silica in the HCPCF. The sharp cutoff at 100 cm^−1^ is from the 785 nm long pass filter. By increasing the pump energy, the 587 cm^−1^ Raman peak intensity increases immensely, and the laser line disappears.

*OPO as the excitation pump:* Figure 2 shows H_2_ Raman spectra for two selected pump energies of 1 and 5 µJ. As shown in Figure 2a, the Raman spectrum contains all four peaks of H_2_ with 1 µJ pump pulses. Increasing the OPO pump pulse energy to 5 µJ results in a huge five orders of magnitude increase in the intensity of the 587 cm^−1^ Raman peak, as shown in Figure 2b.

**Figure 3:**
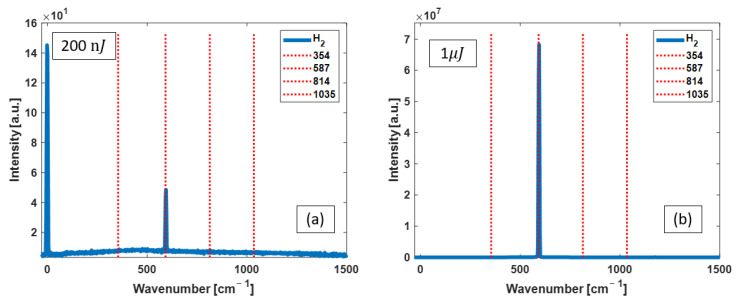
Raman spectra measured with dye laser pulses of 200 nJ (**a**) and 1 μJ (**b**) from the FERS system filled with H_2_. The red dashed lines identify the known peak positions of rotational Raman lines of H_2_. Of note, the pump laser line at 0 cm^−1^ shown in (**a**) diminishes in the spectrum shown in (**b**).

*Dye laser as the excitation pump:* Figure 3 shows the Raman spectra obtained with two pump energies of 200 nJ and 1 µJ. The intensity of the 587 cm^−1^ Raman line increases when pump pulse energy is increased.

### 3.2. CO_2_ Raman Measurements

**Figure 4:**
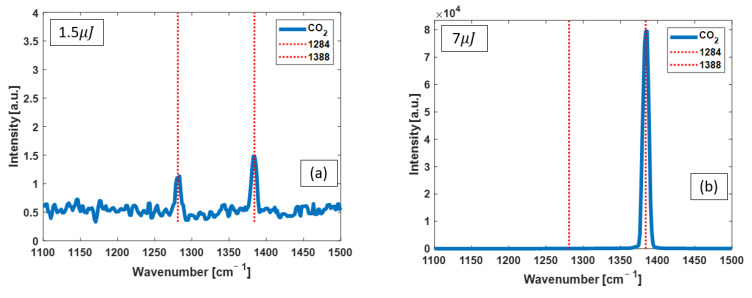
Raman spectra from CO_2_ pumped with OPO pulses of (**a**) 1.5 µJ and (**b**) 7 µJ. The red dashed lines identify the known peak positions of vibrational Raman lines of CO_2_. The other small spikes in the low-energy spectrum are noises. By increasing the pump energy, the intensity of the 1388 cm^−1^ Raman line increases dramatically, and the 1284 cm^−1^ Raman line diminishes.

*OPO as the excitation pump:* The Raman spectra obtained with selected OPO pulses of 1.5 µJ and 7 µJ are shown in Figure 4a and Figure 4b, respectively.

**Figure 5:**
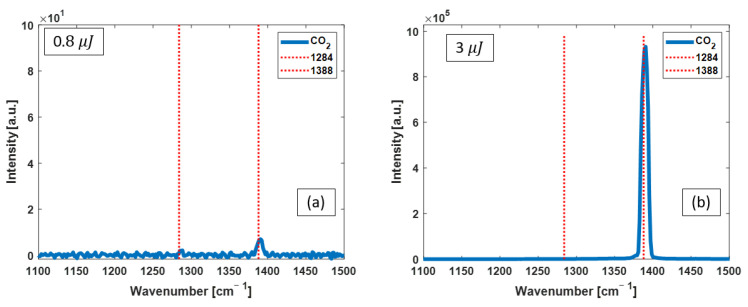
Raman spectra from CO_2_ pumped with dye laser pulses of (**a**) 0.8 µJ and (**b**) 3 µJ. The red dashed lines identify the known peak positions of vibrational Raman lines of CO_2_. The other small spikes in the low-energy spectrum are noises. By increasing the pump energy, the intensity of the 1388 cm^−1^ Raman line increases significantly.

*Dye laser as the excitation pump:* Figure 5 shows the CO_2_ Raman spectra measured with two selected energies of 0.8 µJ and 3 µJ. The significant growth in the 1388 cm^−1^ Raman line is observed with increasing pump energy. This peak was the only signal present and grew in intensity in all the individual spectra obtained with the other pump energies.

### 3.3. Propene Raman Measurements

**Figure 6:**
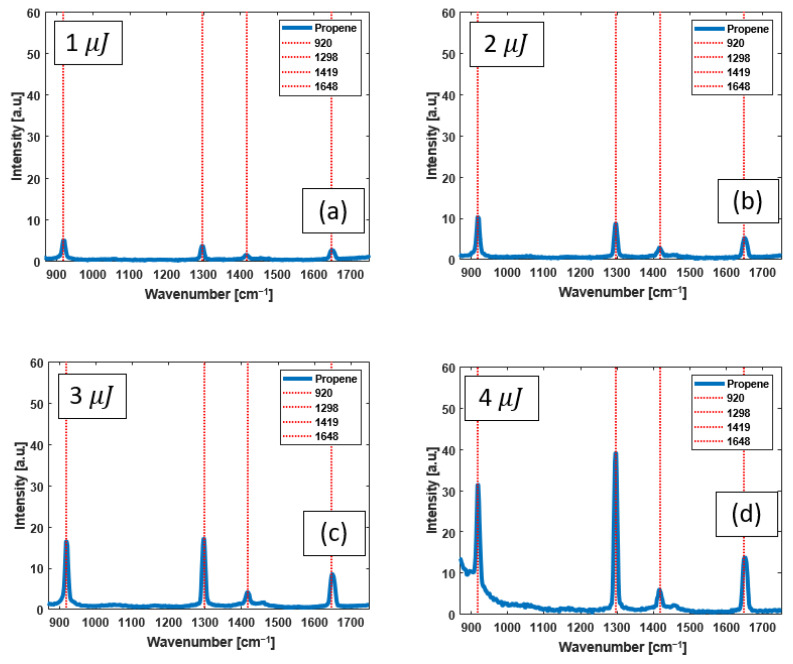
Raman spectra measured from propene pumped with dye laser pulses of (**a**) 1 μJ, (**b**) 2 μJ, (**c**) 3 μJ, and (**d**) 4 μJ. The red dashed lines identify the known peak positions of vibrational Raman lines of propene. The increase in background spectrum in (**d**) is due to Raman scattering in the HCPCF silica material.

Figure 6 shows propene Raman spectra with dye laser pulses with energies from 1 to 4 µJ. As shown, increasing the excitation pulse energy results in an enhanced Raman scattering intensity from all four detected Raman peaks. The increase in the background spectrum of Figure 6d is due to Raman scattering in the HCPCF silica material. By comparing the four individual spectra, it can be seen that the 1298 cm^−1^ Raman peak intensity increases faster than the other three peaks.

### 3.4. Raman Spectral Analysis

The intensity of the Raman lines of the individual spectra obtained from H_2_, CO_2_, and propene were calculated and plotted as a function of pump pulse energy.

**Figure 7:**
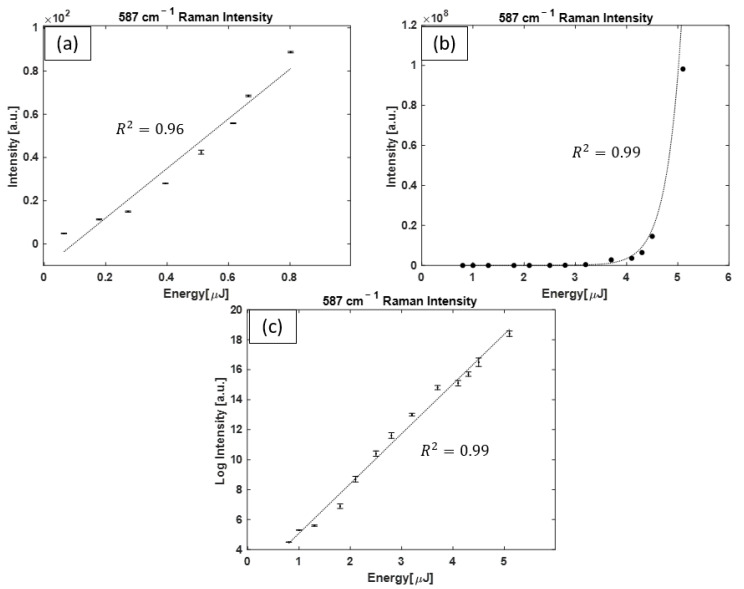
Intensity of the H_2_ Raman peak (587 cm^−1^) as a function of OPO pulse energy. (**a**) The intensity of the 587 ${\mathrm{cm}}^{-1}$ Raman line as a linear function of pump pulse energy, acquired with pump pulses with E ≤ 0.8 µJ, where all Raman peaks grow simultaneously. (**b**) The intensity of the 587 ${\mathrm{cm}}^{-1}$ Raman line as an exponential function of pump pulse energy, acquired with E ≥ 0.8 µJ, where the 587 cm^−1^ Raman line dominantly grows. (**c**) The same data points from (**b**) replotted on a logarithmic–linear scale for a better display resolution of data points. For many data points, the size of the error bars is smaller than the symbols shown.

For H_2_ pumped with OPO pulses, dominant growth in the 587 cm^−1^ peak occurs with pulse energies above ~0.8 µJ. The intensity of the peak for a range of pump energies is plotted in Figure 7a, where a linear function is fitted to the data points (R^2^ = 0.96) with pulse energies ≤ 0.8 µJ. Figure 7b shows the intensity of the Raman peak with pulse energies ≥ 0.8 µJ, where an exponential function is fitted to data points (R^2^ = 0.99). Figure 7c shows the same data points of Figure 7b on a logarithmic–linear scale for a better resolution between data points. As the graphs in Figure 7 display, the trend in the intensity growth with pump energy is complex, and there exists an energy threshold of ~0.8 µJ, where the trend in Raman intensity changes from linear (spontaneous Raman regime) to an exponential function (SRS regime) of the pump pulse energy.

**Figure 8:**
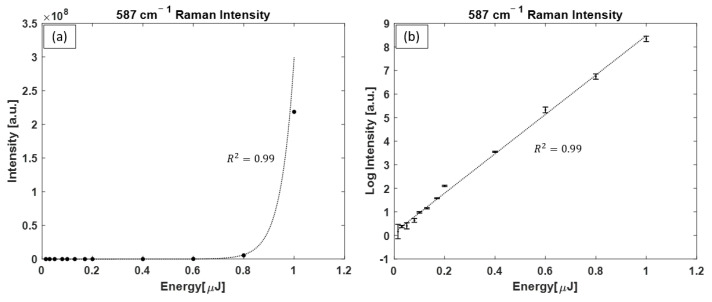
Intensity of the H_2_ Raman peak (587 cm^−1^) as a function of dye laser pulse energy plotted on (**a**) a linear–linear scale with an exponential function fitted to the data points and (**b**) a logarithmic–linear scale plot, where a linear function is fitted to data points. For some data points, the size of the error bars is smaller than the size of the symbols.

Figure 8 displays the intensity of the 587 cm^−1^ Raman peak of H_2_ pumped with the dye laser as a function of the pulse energy. Figure 8a is on a linear–linear scale, where an exponential function with $R^{2}=0.99$ is fitted to data points. Figure 8b shows the same data points on a logarithmic–linear scale with a linear function fitted to data points ($R^{2}=0.99$). Of note, only the 587 cm^−1^ Raman peak was present in all the spectra obtained and, therefore, there was no energy threshold observed for Raman line intensity growth changing from linear to exponential, i.e., the Raman measurements were always in the SRS region for the pulse energy levels used in this experiment due to the high instant intensity levels of the dye laser.

**Figure 9:**
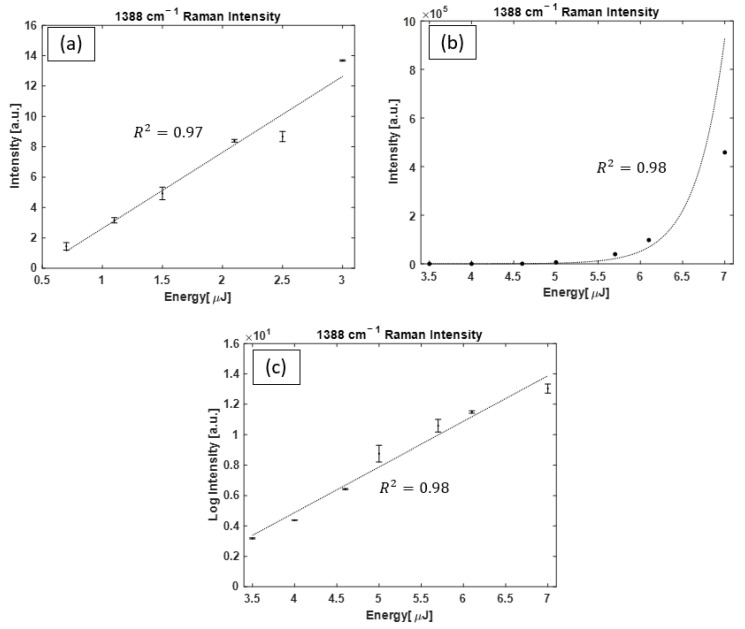
Intensity of the CO_2_ Raman peak (1388 cm^−1^) as a function of OPO pulse pump energy. (**a**) The intensity of the Raman peak obtained with E ≤ 3.5 µJ, with a linear function fitted to the data points showing the trend in Raman line intensity growth. (**b**) The intensity of the Raman peak obtained with E ≥ 3.5 µJ, where an exponential function represents the growth in Raman line intensity with pump pulse energy. (**c**) The same data points from (**b**) plotted on a logarithmic–linear scale for a better display of data point resolution. For some data points, the size of the error bars is smaller than the symbols shown.

A similar type of analysis was performed for CO_2_. With OPO as the pump, when E ≤ 3.5 µJ, both Raman lines grow together and grow in a linear fashion; however, beyond this energy threshold, growth in the 1388 cm^−1^ Raman peak dominates. The evolution of the intensity of this peak as a function of pump energy is shown in Figure 9a for pump pulses with E ≤ 3.5 µJ, with a linear function fitted to data points ($R^{2}=0.97$). Figure 9b shows the intensity of the Raman peak with E ≥ 3.5 µJ, with an exponential function fitted to the data points. Figure 9c shows the same data points as Figure 9b on a logarithmic–linear plot.

**Figure 10:**
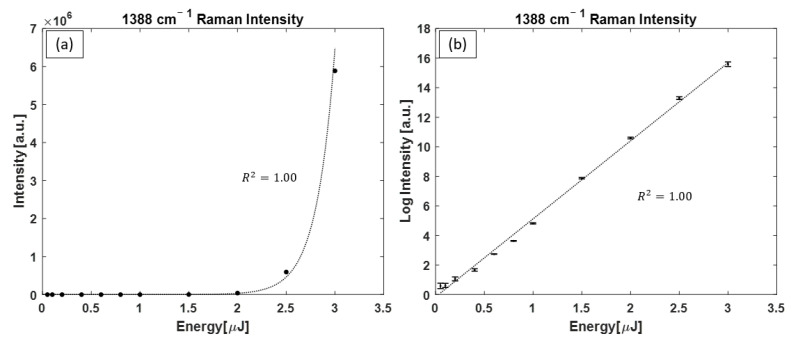
Intensity of the CO_2_ Raman peak (1388 cm^−1^) as a function of dye laser pulsed pump energy. In (**a**), the data points are plotted on a linear–linear axis with an exponential function representing the growth in intensity as a function of pump pulse energy, whereas (**b**) displays the same data points on a logarithmic–linear axis. For many data points, the size of the error bars is smaller than the size of the symbols.

With the dye laser as the pump, the intensity of the 1388 cm^−1^ Raman peaks is plotted in Figure 10a, with an exponential function fitted to data points, and in Figure 10b, with a linear function fitted to data points on logarithmic-linear axes. Note that only the 1388 cm^−1^ Raman peak was present in the acquired spectra.

**Figure 11:**
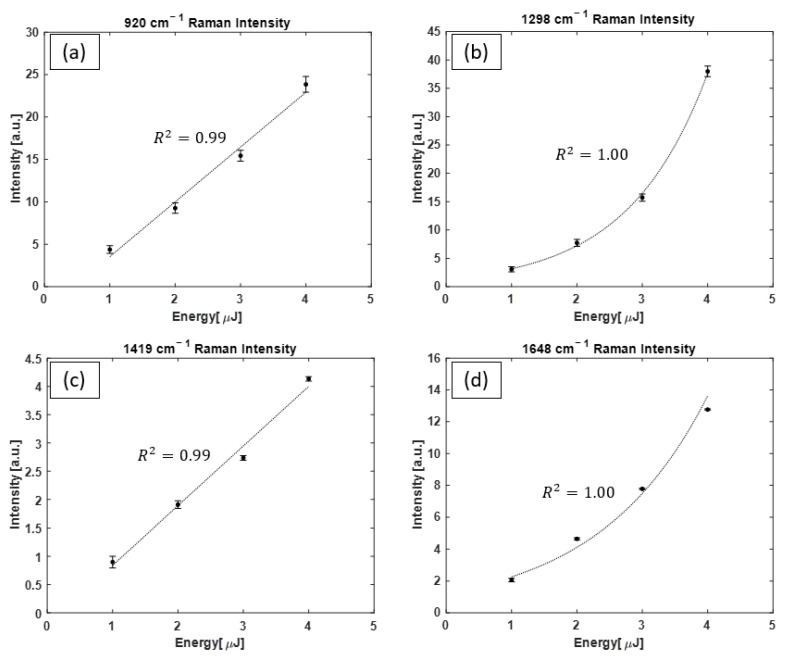
Propene Raman scattering intensity of the (**a**) 920 cm^−1^ peak, (**b**) 1298 cm^−1^ peak, (**c**) 1419 cm^−1^ peak, and (**d**) 1648 cm^−1^ peak as a function of pump dye laser pulse energy. In (**a**,**c**), a linear function fitted to data points shows the linear growth in the 920 cm^−1^ and 1419 cm^−1^ Raman peak intensities, respectively, whereas in (**b**,**d**), an exponential function shows the growth in the intensity of the 1298 cm^−1^ and 1648 cm^−1^ Raman peaks, respectively. For some data points, the size of the error bars is smaller than the size of the symbols.

Figure 11 shows the evolution of propene Raman peaks as a function of the pumping pulse energy. In Figure 11a,c linear function is fitted to data points representing the linear dependence of Raman peak intensity on the pump pulse energy for 920 cm^−1^ and 1419 cm^−1^ bands. However, in Figure 11b,d, an exponential function is fitted to data points, representing the intensity energy dependence that is exponential for 1298 cm^−1^ and 1648 cm^−1^ Raman peaks.

## 4. Discussion

There is an increasing trend in the number of published reports on the application of specialized hollow-core optical fibers for Raman studies [43]. HCPCF results in an enhancement in Raman scattering intensities and therefore is a favorable choice for gas Raman analysis. Several FERS systems have been developed for the purpose of gas Raman analysis with applications in disease diagnosis using Raman analysis of exhaled breath gases, as well as in industrial fields for gas sensing applications. All these FERS systems use CW lasers as the excitation pump for acquiring enhanced spontaneous Raman scattering intensities.

In studies with the goal of developing new laser wavelengths, SRS has been realized in HCPCF filled with gases as an optical gain medium by using a single-beam pulsed pump. New laser lines based on SRS from gas media have been successfully developed.

In the current study, we explored the feasibility of combining a high-energy pulsed laser as the pump for an SRS-capable FERS system for gas analysis purposes for the first time. Acquiring SRS with only a single-beam pump has great advantages as it reduces system complexities and costs when compared with conventional dual-beam pump–Stokes SRS systems.

We carried out measurements with an OPO (pulse width of 7 ns and repetition rate of 20 Hz) and a dye laser (pulse width of 0.8 ns and repetition rate of 7 Hz). At a given pulse energy level, the dye laser has almost an order of magnitude higher power density compared with the OPO because the pulse widths of the two lasers have an 8.8 times difference (0.8 ns/7 ns = 8.8). Using the two lasers in our experiments helped cover a broad light power density range, thus enabling us to observe the transition from spontaneous Raman to SRS.

We studied the trend in Raman scattering intensities by varying the pump pulse energies. Overall, the trend in Raman scattering intensities as a function of pump pulse energy was very complex. Spontaneous Raman scattering intensities vary linearly with pump energies. In the SRS regime, the relationship is no longer linear and is rather exponential. By evaluating the growth of the acquired Raman intensities by gradually varying the pump energies, we aimed to find a region where the Raman scattering intensities deviate from growing linearly with a higher increasing rate. We defined this as the SRS threshold.

Raman measurements of H_2_ and CO_2_ with OPO pump pulses showed that the first four rotational Raman peaks of H_2_ [44,45] and vibrational Raman peaks of CO_2_ [38] are present and grow linearly when smaller pulse energies are used. At higher pulse energies, one Raman peak becomes dominant, and the Raman signals grow exponentially with increasing pulse energy. This represents the transition from spontaneous Raman to SRS. Similar studies with a dye laser showed that there is always only one dominant Raman peak present, and the Raman signal grows exponentially with increasing pulse energy, suggesting that for the whole pulse energy range experimented, the Raman scattering is always in the SRS regime due to the much higher light power density provided by the dye laser.

With the OPO as the excitation pump, the SRS threshold for H_2_ and CO_2_ was 0.8 and 3.5 µJ, equivalent to peak powers of 114 W and 0.5 kW, respectively. One note to consider is that there was no such threshold observed with the range of the energies used to excite Raman with the dye laser as a pump. Considering the different efficiency of coupling with the OPO beam (13%) and the dye laser (30%), as well as the pulse duration of the two lasers (7 ns vs. 0.8 ns), one can estimate the equivalent SRS energy threshold with the dye laser as the pump, i.e., the 0.8 µJ threshold of SRS for H_2_ with the OPO is equivalent to 40 nJ with the dye laser. A similar analogy yields a 3.5 µJ threshold of SRS for CO_2_ with the OPO is equivalent to 170 nJ with the dye laser. As such, in the spectra acquired with the dye laser, we do not have enough low-pulse energy data points to observe the transition from spontaneous Raman to SRS.

Propene has a complex series of Raman–Stokes transitions [46,47], and the four most intense peaks of 920 cm^−1^, 1298 cm^−1^, 1419 cm^−1^, and 1648 cm^−1^ were clearly detected with our system. Examining the Raman spectra of propene suggests the presence of both the spontaneous Raman scattering regime and the SRS regime in the energy range of our performed measurements. This is concluded since a linear trend exists for the growth in intensity of the 920 cm^−1^ and 1419 cm^−1^ Raman lines and an exponential trend for the 1298 cm^−1^ and 1648 cm^−1^ Raman lines. The observed trend is consistent with reports on the Raman scattering spectrum of propene and the differences in the cross section of Raman scattering for different peaks [46,47]. As Lord et al. showed in reference [47], the intensity of the 1298 cm^−1^ and 1648 cm^−1^ Raman peaks is very strong compared with the 920 cm^−1^ and 1419 cm^−1^ Raman peaks, which described the Raman intensity growth observed.

One point to mention regarding propene spectra is the contribution of silica–Raman interactions with the pump, which increases with higher pump energies. This is related to the fact that, unlike H_2_ and CO_2_, where increasing the pump energy results in an SRS of one dominant peak, all four peaks grew in intensity simultaneously, and no dominant SRS peak appeared. Therefore, pump energy loss to propene Stokes gain is not established, and thus, the silica background increases with pump energy.

Propene Raman measurements demonstrated the potential of the FERS system pumped with a single-beam pulsed laser for VOC gas analysis applications in medical and industrial fields. The SRS regime indicated a significant enhancement in Raman scattering intensities, which is crucial for improving the detection sensitivity for Raman-based gas/breath analysis.

## 5. Conclusions

Raman studies with simple and complex gases were performed using an FERS system with a single-beam pulsed laser as the excitation pump, with the aim of realizing SRS for enhanced Raman spectral measurements. Raman spectral measurements were performed with H_2_ and CO_2_ as examples of simple gases and propene as an example of a more complex VOC gas. Our findings showed that a pulsed pump can be utilized in an FERS system to generate SRS for gas analysis. Raman spectral measurements of simple gases showed that there are two regimes of Raman scattering intensity growth as laser pulse energy increases. First, a linear regime is associated with spontaneous Raman scattering, where all the Raman peaks grow together. Second, an SRS regime is associated with an exponential growth in Raman intensity, where one of the peaks grows in intensity significantly faster than other peaks that are present. In gases with much more complex Raman transitions such as propene, both regimes are present. We are actively conducting research in this field to explore the potential of this SRS-capable FERS system in VOC gas analysis with the aim of realizing breath analysis-based lung cancer screening.

## 6. Patents

The BC Cancer Institute filed a provisional patent application related to the work reported this manuscript. The authors may receive royalties from future commercialization of the patent.

## Data Availability

The data are contained within this article.
